# Dust Concentration Forecasting Method for Intermittent Processing of Powder and Granular Materials

**DOI:** 10.3390/s26134207

**Published:** 2026-07-03

**Authors:** Mingming Wang, Zhiyuan Li, Chaobo Li, Xiaoyun Sun, Yi Wang, Zhaofeng He

**Affiliations:** 1 Hebei Provincial Collaborative Innovation Center of Transportation Power Grid Intelligent Integration Technology and Equipment, Shijiazhuang Tiedao University, Shijiazhuang 050043, China; wangmm@stdu.edu.cn; 2 School of Electrical and Electronic Engineering, Shijiazhuang Tiedao University, Shijiazhuang 050043, China; 1202409026@student.stdu.edu.cn (Z.L.); hezhaofeng@stdu.edu.cn (Z.H.); 3 Hebei Academy of Emergency Management Sciences, Shijiazhuang 050031, China; 18630121072@126.com (C.L.); 19931199511@163.com (Y.W.); 4 School of Electronic and Control Engineering, North China Institute of Aerospace Engineering, Langfang 065000, China

**Keywords:** dust concentration forecasting, early warning, iTransformer, DLinear, adaptive gated fusion

## Abstract

Dust concentration during intermittent processing of powder and granular materials is characterized by high-frequency abrupt changes, local accumulation, and complex coupling among multiple sensors. Existing forecasting models still exhibit limitations in modeling global dependencies and characterizing local trends. To address these issues, this paper proposes an iTransformer-based dust concentration forecasting model that integrates a dual-stage feed-forward network and a DLinear branch. With iTransformer as the backbone network, the proposed model captures the coupling relationships among multi-source sensing signals through variate-wise modeling. A progressive dual-stage feed-forward feature refinement mechanism is constructed to enhance the model’s representation capability for transient variations and peak fluctuations in dust concentration. In addition, a collaborative modeling framework consisting of an iTransformer main branch and a DLinear auxiliary branch is designed to jointly learn global nonlinear features and local linear trends. An adaptive gated fusion mechanism is further introduced to dynamically allocate the contribution weights of different branches according to sequential characteristics. Experiments were conducted on a public 1 Hz smoke-sensing dataset, which was used as a proxy benchmark for high-frequency multivariate PM2.5 forecasting rather than direct industrial dust data. Under the setting of a 300-step input length and a 60-step forecasting horizon, the proposed model achieves an MSE of 1.8292 × 10^−3^, an MAE of 0.0334, an RMSE of 0.0428, an MAPE of 0.0177, and an R^2^ of 0.9744, outperforming the compared baseline models in overall performance. The results indicate that the proposed method improves overall forecasting accuracy and provides a methodological reference for sensor-driven particulate concentration forecasting and early warning, while further validation using field data from actual powder and granular material processing workshops is still required before practical deployment.

## 1. Introduction

Intermittent processing of powder and granular materials is a common discrete manufacturing mode in industries such as the chemical industry, pharmaceuticals, and building materials. This type of production process is typically characterized by frequent batch switching, pronounced alternation between process startup and shutdown, and rapid dynamic changes in operating states, making it highly susceptible to generating transient high-concentration dust during stages such as crushing, screening, conveying, and mixing. The rapid accumulation of dust in localized spaces not only increases occupational health risks for operators, but may also lead to safety accidents such as dust explosions [1]. In accordance with GBZ 2.1-2019 Occupational Exposure Limits for Hazardous Agents in the Workplace [2] and GBZ/T 340-2025 Guideline for Prevention and Control of Dust Hazard at Workplace [3], real-time monitoring and early warning of dust concentration are of great significance for improving the intrinsic safety level of production processes.

Unlike the hourly [4] or daily [5] pollution sequences commonly observed in urban atmospheric environments, dust concentration sequences in intermittent processing scenarios typically exhibit high sampling frequency, severe nonlinear fluctuations, frequent abrupt changes, and tight multi-sensor coupling. Particularly under conditions such as equipment startup and shutdown, material impacts, localized disturbances, and operational condition switching, the concentration of fine particulate matter, represented by PM2.5, often experiences rapid surges or significant fluctuations within a short period. Traditional fixed-point monitoring primarily relies on lagging threshold-based alarms, making it difficult to promptly identify concentration evolution trends and provide an effective lead time for early warning. Furthermore, models developed for low-frequency air quality forecasting struggle to fully adapt to industrial dust monitoring scenarios characterized by high-frequency sampling, strong disturbances, and nonstationarity [6,7]. Therefore, short-term multi-step-ahead forecasting of high-frequency particulate matter concentration sequences for early warning holds clear theoretical value and engineering significance.

For PM2.5 concentration forecasting, existing studies have proposed a variety of modeling approaches, including traditional machine learning, deep learning, and self-attention-based methods. Early approaches mainly include Support Vector Regression [8], Random Forest [9], and boosting-based algorithms [10,11]. Although these methods can, to some extent, capture the nonlinear relationships between particulate matter concentration and multi-source influencing factors, they typically rely on manual feature engineering and exhibit limited capability in characterizing complex temporal dependencies and dynamic evolution patterns. Subsequently, deep learning models such as Long Short-Term Memory networks [12] and Convolutional Neural Networks [13] have been widely applied to particulate matter concentration forecasting. Compared with traditional machine learning methods, these models have achieved improvements in temporal modeling and feature extraction. However, they still face challenges such as insufficient capture of long-range dependencies and limited parallel efficiency in long-sequence forecasting tasks.

In recent years, Transformer [14] and its variants have demonstrated strong advantages in multivariate time series forecasting. Models such as Informer [15], Autoformer [16], and iTransformer [17] have enhanced the modeling of global dependencies through self-attention mechanisms. Notably, iTransformer adopts variate-wise modeling and has shown promising performance in multivariate forecasting tasks. It has been applied to gas concentration forecasting [18], PM2.5 concentration forecasting [19], and other multi-step time series forecasting tasks [20,21,22,23]. Meanwhile, linear decomposition models such as DLinear exhibit strong capability in trend extraction and have also shown competitive performance in environmental concentration forecasting [24,25,26]. Recent work has also combined DLinear with Transformer models to capture local trend information and global dependencies in nonstationary time series [27]. Different from these reconstruction or anomaly detection frameworks, this study uses DLinear as an auxiliary branch for PM2.5 forecasting and combines it with an enhanced iTransformer branch through adaptive gated fusion. Although these studies have improved the modeling of particulate matter sequences, several issues remain for PM2.5 forecasting from data sampled at a high frequency. Transformer models still have limited ability to describe local trend changes explicitly, while DLinear is not sufficient for nonlinear fluctuations and abrupt peaks. In addition, the feed-forward module in the standard Transformer can be further improved for finer nonlinear feature refinement. These limitations suggest that a single modeling mechanism is difficult to cover sensor coupling, local trend changes, and abrupt concentration variations at the same time.

The motivation of this study comes from warning of dust risk during intermittent processing of powder and granular materials. Since open datasets collected from actual powder processing workshops at a high sampling frequency are still limited, the experimental validation is conducted on a public smoke-sensing dataset sampled at 1 Hz. This dataset contains PM2.5 and other variables related to particulate matter, as well as several environmental sensor signals. Therefore, it is used in this study as a proxy benchmark for evaluating the forecasting ability of the proposed model on multivariate PM2.5 sequences. The results mainly demonstrate the methodological effectiveness of the model, while further validation with real industrial dust data is still needed before practical deployment.

To address these problems, this study proposes a hybrid forecasting model based on iTransformer. The model uses iTransformer to model relationships among sensor variables, replaces the original feed-forward module with DualStageFFN to refine nonlinear features, and introduces a DLinear branch to provide local trend and fluctuation information. The outputs of the two branches are then combined by an adaptive gate, allowing the model to adjust their contributions under different PM2.5 concentration conditions.

The main contributions of this study are summarized as follows:

(1) A DualStageFFN is introduced into the iTransformer encoder to improve nonlinear feature refinement for high-frequency PM2.5 sequences. Compared with the original feed-forward module, the two stage structure provides a stronger transformation of transient fluctuations and peak-related variations.
(2) A DLinear auxiliary branch is incorporated to complement the iTransformer branch. The iTransformer branch is used to model relationships among sensor variables, while the DLinear branch provides local trend and fluctuation information that is difficult to describe explicitly using attention alone.
(3) An adaptive gated fusion module is designed to combine the two branches under different concentration conditions. Instead of using a fixed weight, the model learns to adjust the branch contributions according to the forecasting context, which improves the stability of forecasting over multiple future steps.

## 2. Methods

**Figure 1:**
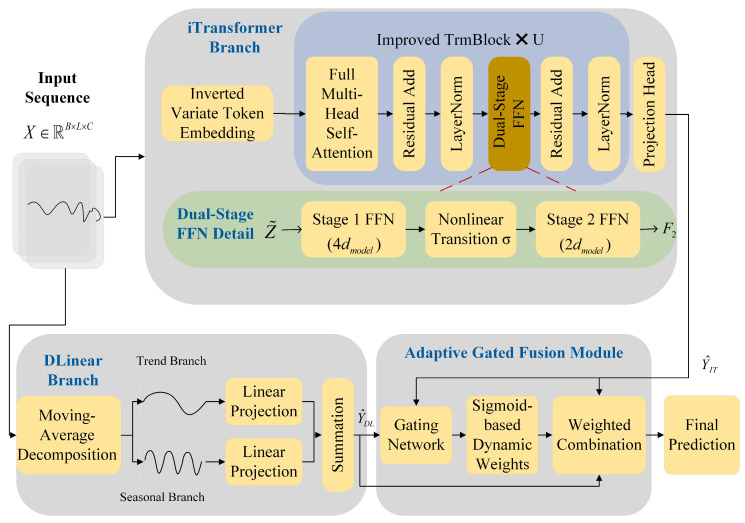
Framework of the proposed hybrid model.

Considering the abrupt variations, coupling among sensor variables, and mixed trend and fluctuation patterns in PM2.5 sequences, this study constructs an iTransformer-based hybrid forecasting model integrating a dual-stage feed-forward network and a DLinear branch. The overall framework is shown in Figure 1, which primarily consists of four core functional modules operating synergistically. First, addressing the multivariate coupling characteristics of the dust data, the model adopts iTransformer as the backbone network and extracts global variable dependencies by transforming the temporal dimension into the variate dimension together with a multi-head self-attention mechanism. Second, targeting the high-frequency abrupt changes induced by intermittent processing, the model introduces a progressive dual-stage feed-forward feature refinement mechanism to achieve the hierarchical refinement and precise capture of nonlinear abrupt signals. Meanwhile, to prevent deep networks from neglecting macroscopic linear cumulative trends, a DLinear auxiliary branch is connected in parallel to provide a robust temporal baseline. Finally, an adaptive fusion gating mechanism is employed to dynamically allocate the output weights of nonlinear abrupt features and linear trend features, thereby achieving synergistic enhancement. The specific internal structures and information transmission mechanisms of each core module are elaborated in detail in the following subsections.

For the convenience of subsequent description, let the multivariate dust time series input to the model be denoted as $X\in {\mathbb{R}}^{B\times L\times C}$ where B, L, C, and P represent the batch size, input sequence length, number of variables, and prediction step length, respectively. The objective of the model is to predict the multivariate output results $\hat{Y}\in {\mathbb{R}}^{B\times P\times C}$ for the future P time steps based on the historical input sequence X.

### 2.1. Multivariate Global Temporal Modeling Based on iTransformer

Within the overall hybrid forecasting framework, the iTransformer backbone branch is used to model global variable dependencies in multivariate dust sequences. Unlike the conventional Transformer, which takes time steps as the modeling units, iTransformer adopts an inverted modeling perspective by transforming the modeling units from temporal tokens to variate tokens, enabling the multi-head self-attention mechanism to capture the correlations between dust concentration and multi-source environmental variables along the variate dimension. Based on this design, the iTransformer backbone branch is divided into three parts, namely inverted variate token embedding, improved encoder layer, and projection forecasting layer, which are responsible for variable representation construction, global dependency modeling, and branch prediction output generation, respectively.

#### 2.1.1. Inverted Variate Token Embedding

For the input sequence defined above, after the dimension inversion it can be represented as $X^{i}\in {\mathbb{R}}^{B\times C\times L}$, where the complete historical sequence of variable m within the input time window is regarded as a variate token and mapped to its initial hidden representation through an embedding operation:

(1) $$e_{m}^{0}=Embedding(X_{:,:,m})$$

where $X_{:,:,m}$ denotes the historical observation sequence of variable m within the input time window; $e_{m}^{0}\in {\mathbb{R}}^{B\times d_{model}}$ represents the initial hidden feature of the variable m after the embedding mapping, and $d_{model}$ denotes the hidden feature dimension after embedding.

By combining the initial hidden features of all variables, the variate token representation input to the improved encoder layer can be obtained:

(2) $$E^{0}=[e_{1}^{0},e_{2}^{0},\ldots ,e_{C}^{0}]$$

where $E^{0}$ denotes the initial feature set composed of all variate tokens, with a dimension of ${\mathbb{R}}^{B\times C\times d_{model}}$.

Through the above inverted variate token embedding process, the original multivariate time series is transformed into a token representation along the variate dimension, enabling the subsequent encoder layers to model the global dependencies between dust concentration and multi-source environmental variables along the variate dimension.

#### 2.1.2. Improved TrmBlock

After the inverted variate token embedding is completed, the obtained initial variate feature set $E^{0}$ is fed into the backbone network composed of U stacked improved TrmBlocks. Its overall update process can be expressed as:

(3) $$E^{l+1}=iTrmBlock(E^{l}),l=0,\ldots ,U-1$$

where iTrmBlock denotes the improved encoder block adopted in this study, U represents the number of stacked encoder blocks, and $E^{l}$ denotes the input feature representation of encoder layer l.

To model the dependency relationships among different variables, the input features are first mapped into a query matrix Q, a key matrix K, and a value matrix V. Subsequently, the correlations among different variables are computed through scaled dot-product attention, and the value vectors are aggregated by weighted summation to obtain the updated feature representations, as formulated in Equation (4).

(4) $$Attention(Q,K,V)=Softmax\left(\frac{QK^{T}}{\sqrt{d_{k}}}\right)V$$

where $K^{T}$ denotes the transpose of matrix K, and $d_{k}$ represents the dimensionality of the key vectors. To enhance the model’s feature extraction capability in different representation subspaces, the multi-head self-attention mechanism is adopted to perform parallel modeling of the above process, and the outputs of all heads are concatenated and then projected to obtain the final result. Through this mechanism, the model can adaptively learn the degree of correlation among different variables, thereby effectively capturing the dependency relationships in multivariate sequences.

After the multi-head self-attention mechanism completes information interaction among variables, the encoder block further enhances its representation capability through a nonlinear feature transformation module. Let $Z^{l}$ denote the feature representation output by the multi-head self-attention sublayer of encoder layer l after residual connection. It is first processed by layer normalization, as formulated in Equation (5).

(5) $${\tilde{Z}}^{l}=LayerNorm(Z^{l})$$

To further enhance the model’s representation capability for complex nonlinear variation features, the conventional feed-forward network in the original iTransformer encoder block is replaced with a progressive dual-stage feed-forward feature refinement module. After the second residual connection and layer normalization, the output of improved encoder layer l can be obtained as:

(6) $$E^{l+1}=LayerNorm\left({\tilde{Z}}^{l}+DualStageFFN\left({\tilde{Z}}^{l}\right)\right)$$

where $DualStageFFN\left({\tilde{Z}}^{l}\right)$ denotes the output of the progressive dual-stage feed-forward feature refinement module. After layer-by-layer feature extraction through U improved encoder layers, the model obtains the final variate-wise feature set $E^{U}$. Through the multi-layer encoder, the model can fully learn the global dependencies between dust concentration and multi-source environmental variables along the variate dimension. The specific structure of the dual-stage feed-forward feature refinement module is further introduced in Section 2.2.

#### 2.1.3. Projection Head

Based on the final variate-wise feature set $E^{U}$ output by the improved TrmBlock, a projection forecasting layer is adopted to map the final hidden representations of all variables into the forecasting space. For variable m, its future forecasting sequence can be expressed as:

(7) $${\hat{Y}}_{IT,m}=Projection(e_{m}^{U})$$

where $e_{m}^{U}$ denotes the final hidden representation of variable m obtained after U improved TrmBlocks, ${\hat{Y}}_{IT,m}$ represents the output of the iTransformer branch for this variable within the forecasting time window, and Projection() denotes the output projection operation. Furthermore, by combining the forecasting results of all variables along the variate dimension, the overall output of the iTransformer backbone branch can be obtained as:

(8) $${\hat{Y}}_{IT}=[{\hat{Y}}_{IT,1},{\hat{Y}}_{IT,2},\ldots ,{\hat{Y}}_{IT,C}]$$

where ${\hat{Y}}_{IT}$ denotes the multivariate forecasting result generated by the iTransformer backbone branch, with a dimension of B×P×C. Through the projection forecasting layer, the global dependency features among variables learned by the encoder are further transformed into forecasting outputs over the future time window, providing the backbone branch output for the subsequent adaptive fusion.

### 2.2. Progressive Dual-Stage Feed-Forward Feature Refinement Mechanism

The feed-forward network in the original iTransformer encoder block typically adopts a single-stage feature transformation structure, which primarily performs nonlinear mapping for each variable token through one stage of dimension expansion and reduction. Although this structure can enhance the feature representation capability to a certain extent, its feature transformation hierarchy remains relatively limited for the complex nonlinear fluctuation patterns in dust concentration sequences. To further improve the feed-forward module’s capability to represent complex temporal patterns while preserving the computational framework of the attention sublayer, this study extends the original single-stage feed-forward network into a hierarchical dual-stage feed-forward feature refinement structure. For simplicity of notation, in the following description of the internal structure of a single improved TrmBlock, the superscript l indicating the encoder layer index is omitted.

Let Z denote the feature representation output by the multi-head self-attention sublayer after residual connection. The normalized feature $\tilde{Z}$ is then fed into the first-stage feed-forward transformation, which can be formulated as:

(9) $$F_{1}=Conv_{1,2}(\sigma (Conv_{1,1}(\tilde{Z})))$$

where $Conv_{1,1}$ and $Conv_{1,2}$ denote the dimension expansion mapping and dimension reduction mapping in the first stage, respectively, and σ represents the nonlinear activation function. According to the model settings, the hidden dimension in the first stage is set to $d_{ff1}=4d_{model}$.

Based on the output of the first stage, the feature is further subjected to nonlinear activation and then fed into the second-stage feed-forward network for another mapping. The computation process can be formulated as:

(10) $$F_{2}=Conv_{2,2}(\sigma (Conv_{2,1}(\sigma (F_{1}))))$$

where $Conv_{2,1}$ and $Conv_{2,2}$ denote the dimension expansion mapping and dimension reduction mapping in the second stage, respectively. The hidden dimension in the second stage is set to $d_{ff2}=2d_{model}$. Corresponding to the overall mapping in Equation (6), the final output of the above progressive dual-stage transformation is given by the second-stage feature.

Finally, the output of the dual-stage feed-forward network $F_{2}$ is combined with its input feature Z through an outer residual connection, followed by another layer normalization operation, to obtain the final output $E^{\prime }$ of the current encoder block:

(11) $$E^{\prime }=LayerNorm(Z+F_{2})$$

Compared with the original single-stage feed-forward network, this structure introduces a continuous nonlinear feature transformation process within the same encoder block, extending the feed-forward mapping from a single transformation to stage-wise feature extraction and progressive feature refinement. Through this design, the model can more fully characterize the complex nonlinear dynamic patterns in dust concentration sequences and enhance the representation capability of the feed-forward module for local fluctuation information and fine-grained variation features. Without changing the computational framework of the attention sublayer, this method achieves a structural enhancement of the feed-forward component in the original iTransformer.

The detailed configuration of the dual-stage feed-forward network used in this study is summarized in Table 1. The kernel size is set to 1 to preserve the variate token modeling structure of iTransformer. GELU is adopted following the original iTransformer encoder and provides a smooth nonlinear mapping for continuous sensor signals.

### 2.3. Primary–Secondary DLinear Auxiliary Branch for Linear Trend Modeling

Considering that the attention-based backbone network is more effective at modeling complex nonlinear dependencies, while its ability to characterize linear trend information in time series is relatively limited, this study introduces DLinear as an auxiliary branch to enhance the model’s capability to represent the linear variation patterns of the trend and seasonal components in the sequence. DLinear decomposes the input representation into a trend component and a seasonal component, performs linear forecasting on them separately, and then sums the two results to obtain the branch output.

The DLinear branch takes the same input sequence as input, and its trend component can then be formulated as:

(12) $$X_{trend}=MovingAvg(X)$$

Correspondingly, the seasonal component can be formulated as:

(13) $$X_{seasonal}=X-X_{trend}$$

where $X_{trend}$ represents the trend term extracted by moving average, and $X_{seasonal}$ indicates the residual component after removing the trend. This study follows the decomposition idea of the trend and seasonal components in DLinear. However, in the scenario of high-frequency dust concentration sequences, this component mainly reflects detrended fluctuation information and local dynamic disturbances, rather than strictly referring to long-period seasonal variations in the conventional sense. On this basis, linear mappings are applied to the trend component and the seasonal component, respectively, and the forecasting process can be formulated as:

(14) $${\hat{Y}}_{trend}=Linear_{trend}(X_{trend})$$

(15) $${\hat{Y}}_{seasonal}=Linear_{seasonal}(X_{seasonal})$$

Finally, the output of the DLinear branch is formulated as:

(16) $${\hat{Y}}_{DLinear}={\hat{Y}}_{trend}+{\hat{Y}}_{seasonal}$$

where ${\hat{Y}}_{trend}$ and ${\hat{Y}}_{seasonal}$ denote the forecasting results of the trend and seasonal components, respectively, and ${\hat{Y}}_{DLinear}$ represents the final output of the DLinear branch. Compared with the backbone network, this branch has a more concise structure and can directly extract linear forecasting information from the sequence decomposition results, thereby providing linear trend information and detrended fluctuation information complementary to the iTransformer backbone for subsequent fusion.

### 2.4. Adaptive Gated Fusion Mechanism

Based on the aforementioned two-branch modeling framework, let the forecasting results of the improved iTransformer main branch with the embedded dual-stage feed-forward network and the DLinear auxiliary branch be denoted by ${\hat{Y}}_{IT}$ and ${\hat{Y}}_{DLinear}$, respectively, where ${\hat{Y}}_{IT},{\hat{Y}}_{DLinear}\in {\mathbb{R}}^{B\times P\times C}$, and P denotes the forecasting horizon. To achieve the adaptive combination of the forecasting information from the two branches, the outputs of the two branches are first concatenated along the variable dimension to obtain the fused representation, which is formulated as:

(17) $$R=Concat({\hat{Y}}_{IT},{\hat{Y}}_{DLinear})$$

where R denotes the fused representation obtained by concatenating the prediction results of the two branches, with a dimensionality of B×P×2C.

Subsequently, R is fed into the gating network to generate the adaptive gating tensor G:

(18) $$G=\sigma (W_{2}\phi (W_{1}R+b_{1})+b_{2})$$

where $W_{1}$ and $W_{2}$ denote the weight matrices of the two fully connected mappings, respectively, $b_{1}$ and $b_{2}$ represent the bias terms, ϕ indicates the GELU activation function, and σ denotes the Sigmoid function. Accordingly, $G\in {\mathbb{R}}^{B\times P\times C}$, and its element values lie within the range of (0, 1).

Finally, the outputs of the two branches are fused by element-wise weighted combination according to the gating weights, yielding the final forecasting result of the model:

(19) $$\hat{Y}=G⊙{\hat{Y}}_{IT}+(1-G)⊙{\hat{Y}}_{DLinear}$$

where ⊙ denotes element-wise multiplication.

The tensor shape transformations in the adaptive gated fusion module are summarized in Table 2.

In the experimental setting, the input length, forecasting horizon, and number of variables are set to 300, 60, and 12, respectively. Therefore, the iTransformer branch and the DLinear branch generate prediction outputs with identical dimensions. After concatenation, the last dimension of the fused representation is doubled because it contains the paired prediction information from the two branches. The gating network subsequently maps this concatenated representation to the same dimension as the final prediction, so that adaptive fusion weights can be assigned to different forecasting steps and variables. In this way, the fusion process is not a fixed weighted average, but a context-dependent branch weighting mechanism that adaptively balances the nonlinear representation from the iTransformer branch and the linear trend information from the DLinear auxiliary branch.

This fusion strategy preserves, within a unified framework, the capability of the improved iTransformer main branch to characterize complex temporal dependency relationships, as well as the capability of the DLinear auxiliary branch to extract linear variation patterns and local fluctuation information. As a result, it enables collaborative modeling of nonlinear dynamic features and linear evolutionary trends in high-frequency dust concentration sequences.

## 3. Experiments and Analysis

### 3.1. Data and Preprocessing

This study conducts experiments using a publicly available high-frequency multivariate sensor dataset [28]. The original dataset contains 62,631 sensor records with a sampling frequency of 1 Hz, and each record is associated with a UTC timestamp. The dataset contains 13 sensor variables: Temperature, Humidity, TVOC, eCO2, Raw H2, Raw Ethanol, Pressure, PM1.0, NC0.5, NC1.0, NC2.5, PM2.5, and Fire Alarm. These variables collectively characterize the dynamic features of environmental states, gas variations, and particulate matter concentrations in high-frequency particulate matter monitoring scenarios. It should be clarified that the dataset used in this study is a publicly available smoke-sensing dataset, rather than data collected from an actual powder and granular material processing workshop. Therefore, this dataset is used as a proxy benchmark for evaluating the proposed model in high-frequency multivariate PM2.5 forecasting. The results mainly demonstrate the methodological effectiveness of the proposed forecasting framework on public sensor data, while its direct generalization to real industrial dust monitoring scenarios still requires further validation using field data.

It should be noted that, in terms of temporal structure, this dataset is not a single continuous sequence, but is composed of five mutually separated continuous sampling segments. The continuous segments were identified according to timestamp continuity, and a temporal gap larger than 1 s was regarded as a discontinuity. There are obvious temporal gaps among different segments, and the PM2.5 concentration levels and fluctuation ranges vary considerably across segments. Considering that multi-step-ahead time series forecasting imposes relatively high requirements on temporal continuity and distribution consistency, this study does not directly merge all segments for modeling. Instead, two continuous segments with the longest lengths were selected as the research objects to ensure sufficient samples for sliding-window forecasting, and 49,989 valid samples are ultimately retained. For the interval region between the two segments, the corresponding samples are explicitly skipped during the sample generation stage to avoid constructing sliding windows across segment boundaries. This ensures that both the input sequences and the forecasting targets are derived from the same continuous observation segment, thereby improving temporal consistency and modeling reliability.

On this basis, the retained records are divided into the training set, validation set, and test set according to a ratio of 7:1:2 in strict chronological order, and no random shuffling is performed before data splitting. To avoid information leakage caused by overlapping sliding windows near subset boundaries, a sample is assigned to a subset only when both its historical input window and future prediction window are completely located within that subset. Samples crossing the boundaries between the training, validation, and test sets are discarded. The training set is used for model parameter learning, the validation set is used for hyperparameter tuning and early stopping control, and the test set is used for final performance evaluation. In this study, the forecasting task is formulated as a multivariate time series forecasting problem. Although the model jointly predicts all 12 sensor variables, PM2.5 is selected as the primary evaluation target because it is directly related to particulate matter concentration and dust-risk warning. Considering that the Fire Alarm variable in the original dataset is mainly used for classification, it is removed from the input features. The remaining 12 variables, namely Temperature, Humidity, TVOC, eCO2, Raw H2, Raw Ethanol, Pressure, PM1.0, NC0.5, NC1.0, NC2.5, and PM2.5, are retained as model inputs, with the historical observations of PM2.5 also included in the input sequence. The normalization parameters are fitted only on the training set and then applied to the validation and test sets, so that information from the validation and test periods is not used during data scaling. The experimental setting uses an input length of 300 steps and a forecasting horizon of 60 steps, that is, the observed sequence over the past 300 s is used to forecast the PM2.5 concentration variation over the next 60 s. It should also be noted that the experimental results in this study mainly reflect the forecasting performance of the model under continuous high-frequency monitoring scenarios with relatively consistent distributions. The generalization capability under extreme high-concentration segments and more complex cross-distribution conditions still requires further investigation.

### 3.2. Experimental Settings

All experiments were implemented using the PyTorch deep learning framework (version 2.0.0, CUDA 11.8) and conducted on a platform equipped with an Intel Xeon Silver 4114 CPU and an NVIDIA GeForce RTX 3090 GPU. During training, the Adam optimizer was adopted for parameter updating, and the configurations of the remaining hyperparameters are listed in Table 3. To ensure a fair comparison, all compared models were evaluated using the same chronological data split, preprocessing procedure, input length, forecasting horizon, and evaluation metrics.

Based on the search ranges listed in Table 3, the hyperparameter configuration of each model was selected according to the validation set performance. For models involving different output strategies, both Type 1 and Type 2 were evaluated on the validation set, and the better performing strategy was fixed for the final test evaluation. The selected learning rate, model dimension, dropout rate, and output strategy of each compared model are summarized in Table 4.

After the selected hyperparameters and output strategy were fixed, each model was independently trained and tested using three random seeds, namely 2021, 2022, and 2023. The final results are reported as mean ± standard deviation over the three runs.

### 3.3. Evaluation Metrics

To comprehensively evaluate the forecasting performance of the proposed model, two groups of metrics are adopted in this study. The first group is used to measure the overall prediction accuracy of PM2.5 concentration, including MSE, MAE, RMSE, MAPE and R^2^. MSE and RMSE reflect the magnitude of forecasting errors and are sensitive to large deviations, MAE represents the average absolute deviation between the predicted values and the true values, offering good interpretability. MAPE characterizes the relative forecasting error, and R^2^ is used to measure the degree to which the model fits the variation trend of the target variable, and a value closer to 1 indicates better fitting performance. These metrics are defined as follows:

(20) $$\mathrm{MSE}=\frac{1}{N}\sum_{i=1}^{N}{\left(y_{i}-{\hat{y}}_{i}\right)}^{2}$$

(21) $$\mathrm{MAE}=\frac{1}{N}\sum_{i=1}^{N}|y_{i}-{\hat{y}}_{i}|$$

(22) $$\mathrm{RMSE}=\sqrt{\frac{1}{N}\sum_{i=1}^{N}{\left(y_{i}-{\hat{y}}_{i}\right)}^{2}}$$

(23) $$\mathrm{MAPE}=\frac{1}{N}\sum_{i=1}^{N}\frac{\left|{\hat{y}}_{i}-y_{i}\right|}{\left|y_{i}\right|+\varepsilon }$$

(24) $$R^{2}=1-\frac{\sum_{i=1}^{N}{\left(y_{i}-{\hat{y}}_{i}\right)}^{2}}{\sum_{i=1}^{N}{\left(y_{i}-\bar{y}\right)}^{2}}$$

where MSE denotes Mean Squared Error, MAE denotes Mean Absolute Error, RMSE denotes Root Mean Squared Error, MAPE denotes Mean Absolute Percentage Error and R^2^ denotes the coefficient of determination. $y_{i}$ denotes the true value, ${\hat{y}}_{i}$ denotes the predicted value, N denotes the number of samples, and $\bar{y}$ denotes the mean of the true values. ε is a small constant used to avoid division by zero in the calculation of MAPE.

In addition to the overall prediction metrics, Warning-oriented metrics are introduced to evaluate model performance during high-concentration periods. Previous studies on extreme value forecasting have shown that average regression errors alone are insufficient to characterize the prediction performance for extreme high or low values [29]. The warning threshold θ is defined as the 90th percentile of PM2.5 values in the training set, so that the threshold is determined without using validation or test information. Five metrics are used for warning evaluation, namely High MAE, Peak Shift Error, Peak Underestimation Rate, False Alarm Rate, and Missed Alarm Rate. For clarity, they are denoted as High MAE, PSE, PUR, FAR, and MAR, respectively. Their definitions are summarized in Table 5. Since the sampling frequency is 1 Hz, one time step in PSE corresponds to one second.

### 3.4. Model Performance Evaluation

To verify the effectiveness of the proposed model in the high-frequency dust concentration forecasting task, this study selects DLinear, Transformer, Informer, Reformer, Flowformer, iTransformer, iInformer, iReformer, and iFlowformer as baseline models. All models are trained and tested under identical conditions in terms of the data splitting strategy, input length, forecasting horizon, and evaluation metrics to ensure the fairness and reliability of the experimental results. Taking the PM2.5 forecasting task with an input length of 300 steps and a forecasting horizon of 60 steps as an example, the forecasting results of different models are analyzed. The average results of different models on the test set are presented in Table 6.

As shown in Table 6, the proposed adaptive fusion model achieves the best overall forecasting performance among all compared models. It obtains the lowest MSE of (1.8292 ± 0.1353) × 10^−3^, MAE of 0.0334 ± 0.0010, RMSE of 0.0428 ± 0.0016, and MAPE of 0.0177 ± 0.0005, as well as the highest R^2^ of 0.9744 ± 0.0019. These results indicate that the proposed model achieves superior overall prediction accuracy and fitting capability under the setting of an input length of 300 and a prediction horizon of 60 steps. The relatively small standard deviations over three random seeds further suggest that the proposed model maintains stable performance under different random initializations.

Among the baseline models, the inverted structure models generally achieve better forecasting performance than the conventional Transformer-based models. In particular, iReformer shows the strongest performance among the baselines, with an MSE of (2.5258 ± 0.0499) × 10^−3^, an MAE of 0.0392 ± 0.0005, an RMSE of 0.0503 ± 0.0005, an MAPE of 0.0207 ± 0.0003, and an R^2^ of 0.9647 ± 0.0007. Based on the mean values, the proposed adaptive fusion model reduces MSE, MAE, RMSE, and MAPE by 27.58%, 14.80%, 14.91%, and 14.49%, respectively, compared with iReformer. Compared with the original iTransformer backbone, the corresponding reductions are 31.08%, 15.87%, 16.89%, and 16.11%, respectively, while R^2^ increases from 0.9629 ± 0.0030 to 0.9744 ± 0.0019.

The standalone DLinear model obtains an MSE of (55.5129 ± 0.0679) × 10^−3^, an MAE of 0.1813 ± 0.0003, and an R^2^ of 0.2233 ± 0.0009, indicating that a purely linear decomposition model is insufficient for this high-frequency PM2.5 forecasting task. However, when DLinear is used as an auxiliary branch in the proposed hybrid framework, it provides complementary local trend information to the nonlinear iTransformer backbone. Therefore, the improvement of the proposed model can be attributed to the coordinated modeling of multivariate dependencies, dual-stage nonlinear feature refinement, and auxiliary local trend compensation.

As shown in Table 7, the proposed adaptive fusion model exhibits favorable warning-related performance during high-concentration periods. It achieves the lowest High MAE of 0.0457 ± 0.0040 and the lowest PSE of 2.7002 ± 0.2373 among all compared models. This indicates that the proposed model not only reduces the prediction error in high-concentration regions, but also improves the temporal localization of peak events. Compared with the original iTransformer backbone, the High MAE decreases from 0.0542 ± 0.0048 to 0.0457 ± 0.0040, corresponding to a reduction of 15.68%. Meanwhile, the PSE decreases from 2.9236 ± 0.1402 to 2.7002 ± 0.2373. These results suggest that the adaptive fusion strategy enhances the model sensitivity to high-concentration variations while maintaining stable peak timing estimation.

The FAR of the proposed model is 29.2778 ± 3.7516 × 10^−4^, which is lower than those of iTransformer, iInformer, iReformer, and iFlowformer. This suggests that the improvement in high-concentration prediction is not obtained by simply increasing the number of warning predictions. In contrast, although the conventional Transformer model obtains an extremely low FAR, its MAR reaches 0.7644 ± 0.0292, indicating that most true high-concentration points are not detected. Therefore, a low FAR alone does not necessarily indicate reliable warning performance. Informer, Reformer, Flowformer, and DLinear also show substantially larger High MAE, PSE, and MAR values, reflecting their limited ability to capture abrupt high-concentration changes. It should be noted that the PUR of the proposed model is not the lowest among all models, suggesting that peak underestimation still exists in some cases. Nevertheless, considering its lowest High MAE and PSE together with relatively low FAR and MAR, the proposed adaptive fusion model achieves a more balanced performance in high-concentration warning evaluation.

**Figure 2:**
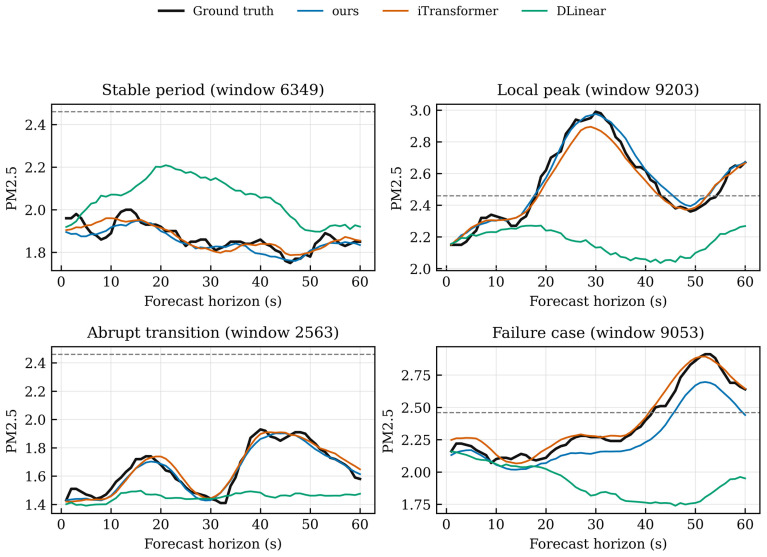
Warning-oriented performance of different models under high-PM2.5 conditions. The dashed horizontal line indicates the warning threshold θ.

To further examine the above warning-related behavior visually, a multi-case forecasting comparison is provided in Figure 2, including low concentration, local peak, abrupt transition, and failure cases. For clarity, only the proposed model, iTransformer, and DLinear are shown. The proposed model generally follows the ground truth more closely than the compared models under stable, local peak, and abrupt transition conditions. In the stable period, the proposed model maintains a smooth prediction and avoids the obvious overestimation observed in DLinear. In the local peak and abrupt transition cases, it better captures the main rising and falling trends, indicating improved adaptability to high-concentration variations. However, the failure case shows that the proposed model still underestimates the amplitude of some sharp peaks. This observation is consistent with the warning-related results and indicates that peak amplitude preservation remains a limitation of the current model.

### 3.5. Performance Under Different Forecasting Horizons

To further evaluate the performance of the proposed model under different forecasting horizons, this study fixes the input sequence length at 300 steps and sets the forecasting horizons to 30, 60, 96, 180, and 300 steps, respectively, thereby constructing five forecasting tasks: 300-30, 300-60, 300-96, 300-180, and 300-300. Except for the forecasting horizon, the data partitioning strategy, model architecture, and major hyperparameter settings remain identical across all experimental groups to ensure the comparability of different forecasting tasks. The experimental results are presented in Table 8 and Table 9.

As shown in Table 8 and Table 9, the forecasting and warning performance of the proposed model changes regularly with the increase in the forecasting horizon. For the forecasting metrics, MSE increases from 1.4467 × 10^−3^ to 5.7816 × 10^−3^, MAE increases from 0.0294 to 0.0593, and R^2^ decreases from 0.9797 to 0.9197 as the forecasting horizon increases from 30 to 300 steps. This indicates that longer horizon forecasting introduces greater uncertainty and increases the difficulty of accurately modeling future PM2.5 variations. A similar trend can also be observed in the warning-related metrics. High MAE increases from 0.0372 to 0.0942, PSE increases from 1.6023 to 10.1461, and MAR increases from 0.1134 to 0.3428, indicating that peak value estimation, peak timing localization, and high-concentration detection become more challenging under longer forecasting horizons. Nevertheless, the model maintains relatively low errors and high R^2^ values under short and medium forecasting horizons, suggesting that the proposed method has stable forecasting and warning capability within a moderate prediction range.

### 3.6. Ablation and Interpretability Analysis

To verify the contribution of each proposed improvement module to the enhancement of model performance, this study conducts ablation experiments based on the iTransformer backbone framework. Specifically, six models are constructed, including the original iTransformer, the iTransformer with the dual-stage feed-forward network, the iTransformer with the DLinear branch and fixed weight fusion, the iTransformer with the DLinear branch and adaptive gated fusion, the model simultaneously introducing the dual-stage feed-forward network and the DLinear branch with fixed-weight fusion, and the complete model with the adaptive gated fusion mechanism. All models are trained and tested under identical conditions regarding the data partitioning strategy, input–output settings, and evaluation metrics. The experimental results are presented in Table 10.

As shown in Table 10, the proposed model achieves the best overall forecasting performance among all ablation variants. Compared with the original iTransformer, the proposed model reduces the MSE from 2.6539 × 10^−3^ to 1.8292 × 10^−3^, while the MAE, RMSE, and MAPE are also reduced from 0.0397, 0.0515, and 0.0211 to 0.0334, 0.0428, and 0.0177, respectively. Meanwhile, R^2^ increases from 0.9629 to 0.9744. These results indicate that the proposed hybrid structure effectively improves the overall PM2.5 forecasting accuracy.

The contribution of each module can be further observed from the intermediate variants. After replacing the original feed-forward network with the DualStageFFN, the MSE decreases from 2.6539 × 10^−3^ to 2.0363 × 10^−3^, indicating that the dual-stage nonlinear feature refinement enhances the representation capability of the iTransformer backbone. When only the DLinear branch is introduced, the MSE decreases to 2.5250 × 10^−3^ under fixed fusion and to 2.3775 × 10^−3^ under adaptive fusion. This suggests that the DLinear branch can provide complementary trend information, but its independent contribution is relatively limited compared with the DualStageFFN. When both DualStageFFN and DLinear are used, the fixed fusion variant obtains an MSE of 1.9033 × 10^−3^, and the complete adaptive fusion model further reduces it to 1.8292 × 10^−3^. Therefore, the final improvement is not caused by a single module, but by the combined effect of nonlinear feature refinement, auxiliary linear trend modeling, and adaptive branch weighting.

Table 11 further compares the ablation variants from the perspective of warning-oriented forecasting performance. The results show that different modules have different effects on high-concentration prediction and threshold-based warning reliability. Compared with the original iTransformer, the DualStageFFN variant reduces High MAE from 0.0542 to 0.0444 and decreases MAR from 0.2014 to 0.1430, indicating that the enhanced nonlinear backbone improves the fitting ability for high-concentration periods and helps reduce missed alarms. However, its FAR increases from 36.5785 × 10^−4^ to 41.0063 × 10^−4^, suggesting that stronger sensitivity to high-concentration points may also introduce more false alarms.

The variants with only the DLinear branch do not consistently improve the warning-oriented metrics. In particular, the fixed fusion variant without DualStageFFN increases High MAE and FAR, while the adaptive fusion variant shows higher PUR and MAR than the original iTransformer. This indicates that linear trend compensation alone is insufficient for reliable high-concentration warning. After combining DualStageFFN and DLinear, the fixed fusion variant achieves the lowest High MAE, whereas the proposed adaptive fusion model achieves the lowest FAR. These results suggest that fixed fusion is more conservative in fitting high-concentration points, while adaptive fusion provides better control of false alarms. Overall, the proposed model achieves the best overall forecasting accuracy and a more balanced warning performance, although peak underestimation and missed alarms remain limitations that require further improvement.

To further explain the internal mechanism behind the ablation results, feature representation visualization and adaptive gate weight analysis are conducted in the following part. The feature map comparison is used to examine how the DualStageFFN changes the representation of particulate-related variables, while the gate weight distribution is used to analyze how the model balances the iTransformer branch and the DLinear branch under different PM2.5 regimes.

**Figure 3:**
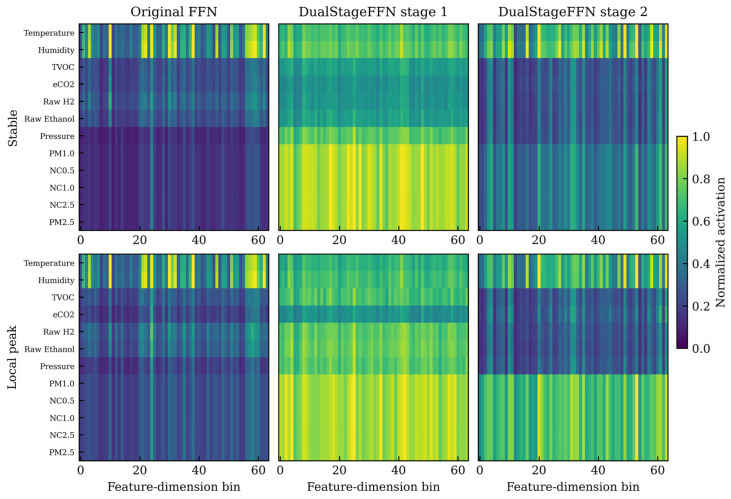
Dual-stage FFN feature visualization.

Figure 3 presents the feature representations extracted by the conventional FFN and the two stages of the proposed DualStageFFN. The complete feature map includes all sensor variables, while the analysis focuses on particulate related variables because PM2.5 is the primary forecasting target in this study. Compared with the conventional FFN, Stage 1 produces broader activation responses across feature dimensions, indicating that the expanded hidden representation enhances the feature extraction capacity of the backbone network. Stage 2 further refines these responses and makes the representation more concentrated on particulate-related features. The peak to stable activation ratio increases from 1.17 after Stage 1 to 1.64 after Stage 2, suggesting that the second stage further strengthens the representation of peak-related features rather than simply repeating the first transformation. This explains why the DualStageFFN improves the overall forecasting performance and the high-concentration prediction performance in the ablation results.

**Figure 4:**
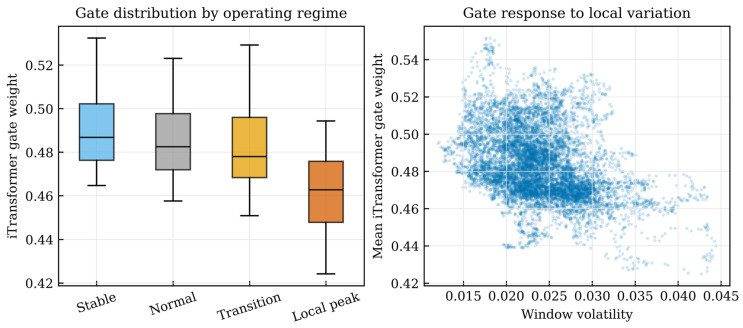
Adaptive gate distribution.

Figure 4 shows the distribution of adaptive gate weights under different PM2.5 conditions. In the proposed fusion formulation, a larger gate value indicates a higher contribution from the iTransformer branch, whereas a smaller gate value indicates a higher contribution from the DLinear branch. The PM2.5 gate has a mean value of 0.4619, a standard deviation of 0.0233, and a range of 0.3458 to 0.6315, indicating that the fusion weight does not collapse to a fixed coefficient. The mean gate decreases from 0.4694 in stable windows to 0.4533 in transition windows, and from 0.4650 in normal concentration windows to 0.4416 in local peak windows. This indicates that the model assigns relatively more weight to the DLinear auxiliary branch during high-concentration and high-variation periods. Therefore, the adaptive gate works as a context-dependent branch balancing mechanism, which helps the model combine nonlinear dependency modeling and local trend compensation.

### 3.7. Inference Time

To further verify the real-time applicability of the proposed model in high-frequency dust concentration early warning scenarios, the inference time of the optimal model is evaluated in this study. Under the setting of an input length of 300, a forecasting horizon of 60, and 9938 test samples, the total inference time on the test set is recorded, and the average forward inference time for each sample window is calculated. The experiments are conducted on an NVIDIA GeForce RTX 3090 GPU and an Intel Xeon Silver 4114 CPU, and the results are presented in Table 12.

As shown in Table 5, the proposed model achieves an average inference time of 6.2947 ms per sample in the GPU environment, with a total forward inference time of 66.0959 s for 9938 test samples. In the CPU environment, the average inference time per sample is 17.2599 ms, and the total forward inference time is 171.9905 s. Since the sampling frequency of the data used in this study is 1 Hz, the interval between two consecutive data updates is 1 s. The time required by the model to complete one forecasting process with 300 input steps and 60 output steps in both the CPU and GPU environments is significantly shorter than 1 s. This indicates that, while maintaining high forecasting accuracy, the proposed model also possesses strong real-time inference capability and can satisfy the timeliness requirements of high-frequency dust concentration monitoring and early warning. In addition, a single forecasting task can still be completed within the millisecond range in the CPU environment, indicating that the proposed model is suitable not only for high-performance GPU computing platforms, but also has potential for edge deployment and industrial field applications.

## 4. Conclusions

Focusing on high-frequency particulate concentration forecasting with abrupt fluctuations, local accumulation, and tight multivariate sensor coupling, this study proposes an iTransformer-based dust concentration forecasting model integrating a dual-stage feed-forward network and a DLinear branch. The proposed model adopts iTransformer as the backbone network and captures the coupling relationships among multi-source sensing variables through variate-wise modeling. A progressive dual-stage feed-forward network structure is introduced to enhance the model’s representation capability for complex nonlinear features, local abrupt changes, and peak fluctuations. In addition, a collaborative modeling framework consisting of an iTransformer main branch and a DLinear auxiliary branch is constructed to achieve the joint learning of global nonlinear patterns and local linear trends. Furthermore, an adaptive gated fusion mechanism is employed to dynamically allocate the contribution weights of different branches according to sequence variation characteristics, thereby improving the forecasting adaptability of the model in high-frequency, strongly disturbed dust concentration sequences.

The experimental results demonstrate that the proposed model achieves superior overall forecasting performance compared with the baseline models and ablation variants. With an input length of 300 time steps and a forecasting horizon of 60 time steps, the proposed model obtains an MSE of 1.8292 × 10^−3^, an MAE of 0.0334, an RMSE of 0.0428, an MAPE of 0.0177, and an R^2^ of 0.9744. The ablation results indicate that the performance improvement is not attributable to a single component, but results from the combined effect of progressive nonlinear feature refinement, auxiliary linear trend modeling, and adaptive branch weighting. In addition to conventional regression metrics, warning-oriented indicators were introduced to evaluate forecasting behavior during high-concentration periods. The proposed model achieves the lowest false alarm rate among the ablation variants, suggesting that the adaptive fusion mechanism contributes to reducing unnecessary alarms in threshold-based monitoring. Nevertheless, the model does not achieve the optimum value for all warning-oriented metrics, and peak underestimation and missed alarms are still observed in some high-concentration windows, indicating that further improvement is required for peak sensitive forecasting and warning reliability.

From an application perspective, the proposed method can provide a methodological reference for sensor-driven short-term particulate concentration forecasting and early warning. In practical industrial monitoring, such a forecasting module has the potential to be integrated with ventilation control, dust removal equipment, personal protection reminders, or low-energy environmental intervention systems to support proactive risk management [30]. Nevertheless, the experiments in this study were conducted on a public high-frequency smoke-sensing dataset, which was used as a proxy benchmark rather than direct industrial dust data. Therefore, the results cannot be directly generalized to actual powder and granular material processing workshops without further validation.

## Figures and Tables

**Table 1:** Architectural configuration of the dual-stage feed-forward feature module.

Module	Dimension Transformation	Kernel Size	Activation
Stage 1	512 → 2048 → 512	1	GELU
Stage 2	512 → 1024 → 512	1	GELU

**Table 2:** Tensor shape transformations in the adaptive gated fusion module.

Tensor	Shape	Description
X	B × 300 × 12	Input sequence
${\hat{Y}}_{IT}$	B × 60 × 12	iTransformer output
${\hat{Y}}_{DLinear}$	B × 60 × 12	DLinear output
R	B × 60 × 24	Concatenated output
G	B × 60 × 12	Gating weight
$\hat{Y}$	B × 60 × 12	Final prediction

**Table 3:** Experimental settings and hyperparameter search ranges.

Hyper-Parameters	Values
Epochs	10
Batch size	32
Number of heads	8
Input sequence length	300
Early stopping patience	3
Number of encoder layers	2
Model dimension search range	{256, 512}
Dropout search range	{0.03, 0.05, 0.1}
Learning rate search range	{0.0001, 0.0003, 0.0005}
Random seeds	{2021, 2022, 2023}

**Table 4:** Selected hyperparameters and output strategies of compared models.

Model	Learning Rate	d_model/moving_avg	Dropout	Output Strategy
DLinear	0.0003	25	Not applicable	Not applicable
Transformer	0.0003	512	0.03	Type1
Informer	0.0003	512	0.03	Type2
Reformer	0.0001	512	0.05	Type1
Flowformer	0.0003	512	0.03	Type1
iTransformer	0.0003	512	0.03	Type1
iInformer	0.0003	512	0.03	Type1
iReformer	0.0003	512	0.03	Type1
iFlowformer	0.0005	512	0.03	Type1
Ours	0.0003	512	0.03	Type1

**Table 5:** Definitions of warning-related evaluation metrics.

Metric	Definition
High MAE	MAE calculated over prediction points whose true PM2.5 values exceed the warning threshold.
PSE	Average time step deviation between the predicted peak position and windows whose true peak exceeds the threshold.
PUR	Proportion of high-peak windows in which the predicted peak value is lower than the true peak value.
FAR	Proportion of normal prediction points that are incorrectly predicted as high-concentration points.
MAR	Proportion of true high-concentration points that are not detected by the prediction.

**Table 6:** Performance comparison of different models on the PM2.5 forecasting task.

Models	MSE (×10^−3^)	MAE	RMSE	MAPE	R^2^
iTransformer	2.6539 ± 0.2138	0.0397 ± 0.0014	0.0515 ± 0.0021	0.0211 ± 0.0008	0.9629 ± 0.0030
iInformer	2.6676 ± 0.1264	0.0404 ± 0.0010	0.0516 ± 0.0012	0.0214 ± 0.0005	0.9627 ± 0.0018
iReformer	2.5258 ± 0.0499	0.0392 ± 0.0005	0.0503 ± 0.0005	0.0207 ± 0.0003	0.9647 ± 0.0007
iFlowformer	3.0195 ± 0.1929	0.0417 ± 0.0016	0.0549 ± 0.0018	0.0222 ± 0.0008	0.9578 ± 0.0027
Informer	60.1583 ± 8.3646	0.1893 ± 0.0132	0.2449 ± 0.0168	0.0963 ± 0.0056	0.1583 ± 0.1170
Reformer	57.1163 ± 10.7000	0.1846 ± 0.0186	0.2383 ± 0.0219	0.0938 ± 0.0079	0.2008 ± 0.1496
Flowformer	45.2819 ± 4.7020	0.1590 ± 0.0094	0.2126 ± 0.0112	0.0805 ± 0.0039	0.3664 ± 0.0658
Transformer	28.7106 ± 4.9370	0.1240 ± 0.0140	0.1690 ± 0.0150	0.0622 ± 0.0065	0.5983 ± 0.0691
DLinear	55.5129 ± 0.0679	0.1813 ± 0.0003	0.2356 ± 0.0001	0.0957 ± 0.0002	0.2233 ± 0.0009
Ours	1.8292 ± 0.1353	0.0334 ± 0.0010	0.0428 ± 0.0016	0.0177 ± 0.0005	0.9744 ± 0.0019

**Table 7:** Comparison of warning-related metrics for different models.

Models	High MAE	PSE	PUR	FAR (×10^−4^)	MAR
iTransformer	0.0542 ± 0.0048	2.9236 ± 0.1402	0.7843 ± 0.0574	36.5785 ± 5.1519	0.2014 ± 0.0241
iInformer	0.0564 ± 0.0051	3.4102 ± 0.5292	0.7794 ± 0.0046	40.7635 ± 10.8534	0.2199 ± 0.0415
iReformer	0.0553 ± 0.0019	2.9652 ± 0.3517	0.7079 ± 0.0540	51.2087 ± 6.0334	0.1706 ± 0.0150
iFlowformer	0.0548 ± 0.0016	3.4043 ± 0.6145	0.8112 ± 0.0051	35.2836 ± 4.0896	0.2068 ± 0.0245
Informer	0.5466 ± 0.0460	19.2585 ± 2.2137	0.9910 ± 0.0155	9.4972 ± 12.7195	0.9117 ± 0.0924
Reformer	0.4821 ± 0.0560	19.8921 ± 1.6733	0.9572 ± 0.0204	9.0001 ± 7.0959	0.9134 ± 0.0338
Flowformer	0.4617 ± 0.0137	15.0033 ± 1.0912	0.9903 ± 0.0124	6.2024 ± 7.5776	0.8714 ± 0.0380
Transformer	0.3553 ± 0.0378	9.9777 ± 1.2543	0.9997 ± 0.0004	0.2254 ± 0.1201	0.7644 ± 0.0292
DLinear	0.3626 ± 0.0014	20.2185 ± 0.1521	0.8255 ± 0.0015	102.0185 ± 1.9579	0.7254 ± 0.0056
Ours	0.0457 ± 0.0040	2.7002 ± 0.2373	0.8611 ± 0.0429	29.2778 ± 3.7516	0.1876 ± 0.0315

**Table 8:** Forecasting performance under different prediction horizons.

Forecasting Horizons	MSE (×10^−3^)	MAE	RMSE	MAPE	R^2^
30	1.4467	0.0294	0.0380	0.0156	0.9797
60	1.7531	0.0328	0.0419	0.0174	0.9755
96	2.0411	0.0356	0.0452	0.0189	0.9715
180	3.1579	0.0444	0.0562	0.0236	0.9560
300	5.7816	0.0593	0.0760	0.0314	0.9197

**Table 9:** Warning-related performance under different prediction horizons.

Forecasting Horizons	High MAE	PSE	PUR	FAR (×10^−4^)	MAR
30	0.0372	1.6023	0.7443	36.6836	0.1134
60	0.0411	2.5765	0.8117	33.3299	0.1607
96	0.0449	2.7935	0.8232	37.5544	0.1688
180	0.0543	4.3105	0.9092	41.0215	0.1819
300	0.0942	10.1461	0.9743	33.3732	0.3428

**Table 10:** Ablation study of overall forecasting performance.

Models	MSE (×10^−3^)	MAE	RMSE	MAPE	R^2^
iTransformer	2.6539 ± 0.2138	0.0397 ± 0.0014	0.0515 ± 0.0021	0.0211 ± 0.0008	0.9629 ± 0.0030
iTransformer + Dual-FFN	2.0363 ± 0.1452	0.0353 ± 0.0013	0.0451 ± 0.0016	0.0187 ± 0.0007	0.9715 ± 0.0020
iTransformer + DLinear + Fixed Fusion	2.5250 ± 0.1900	0.0388 ± 0.0014	0.0502 ± 0.0019	0.0205 ± 0.0008	0.9647 ± 0.0027
iTransformer + DLinear + adaptive Fusion	2.3775 ± 0.1809	0.0376 ± 0.0013	0.0487 ± 0.0019	0.0199 ± 0.0007	0.9667 ± 0.0025
iTransformer + Dual-FFN + DLinear + Fixed Fusion	1.9033 ± 0.0866	0.0341 ± 0.0006	0.0436 ± 0.0010	0.0181 ± 0.0003	0.9734 ± 0.0012
Ours	1.8292 ± 0.1353	0.0334 ± 0.0010	0.0428 ± 0.0016	0.0177 ± 0.0005	0.9744 ± 0.0019

**Table 11:** Ablation study of warning-oriented forecasting performance.

Models	High MAE	PSE	PUR	FAR (×10^−4^)	MAR
iTransformer	0.0542 ± 0.0048	2.9236 ± 0.1402	0.7843 ± 0.0574	36.5785 ± 5.1519	0.2014 ± 0.0241
iTransformer + Dual-FFN	0.0444 ± 0.0033	2.6121 ± 0.2806	0.7264 ± 0.0121	41.0063 ± 2.2990	0.1430 ± 0.0141
iTransformer + DLinear + Fixed Fusion	0.0577 ± 0.0093	2.8560 ± 0.3078	0.7686 ± 0.0280	43.1392 ± 9.3513	0.1944 ± 0.0626
iTransformer + DLinear + adaptive Fusion	0.0546 ± 0.0091	3.0013 ± 0.3152	0.8237 ± 0.0720	36.3646 ± 7.8227	0.2032 ± 0.0294
iTransformer + Dual-FFN + DLinear + Fixed Fusion	0.0423 ± 0.0014	2.7932 ± 0.0800	0.7689 ± 0.0112	38.6478 ± 2.4591	0.1578 ± 0.0034
Ours	0.0457 ± 0.0040	2.7002 ± 0.2373	0.8611 ± 0.0429	29.2778 ± 3.7516	0.1876 ± 0.0315

**Table 12:** Inference time of the proposed model.

Device	Average Inference Time per Sample (ms)	Standard Deviation (ms)	Total Inference Time on the Test Set (s)
NVIDIA GeForce RTX 3090 GPU	6.2947	6.9513	66.0959
Intel Xeon Silver 4114 CPU	17.2599	3.3042	171.9905

## Data Availability

The dataset used in this study is publicly available as cited in Ref. [28]. Further inquiries can be directed to the corresponding author.
